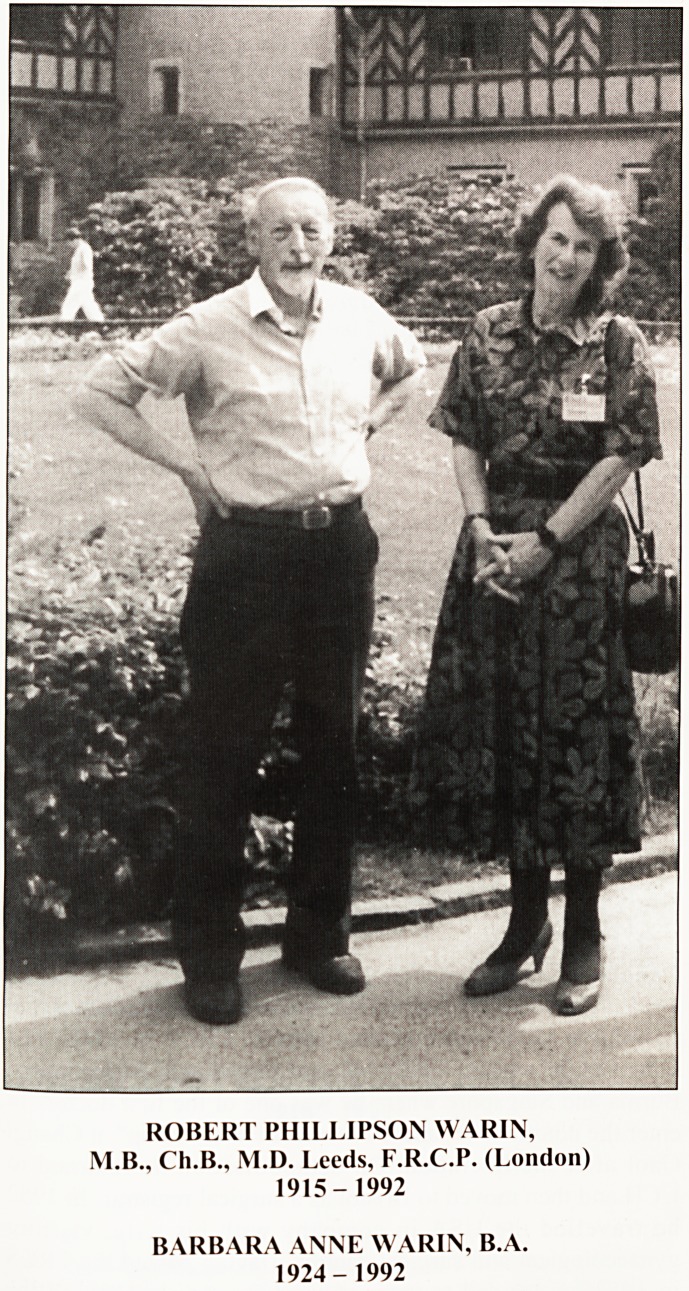# Dr R P Warin

**Published:** 1992-12

**Authors:** J. L. Burton


### Obituary

**ROBERT PHILLIPSON WARIN,**
**M.B., Ch.B., M.D. Leeds, F.R.C.P. (London)**
**1915 – 1992**

**BARBARA ANNE WARIN, B.A.**
**1924 – 1992**

British dermatology and the Bristol medical community suffered a grievous loss on 1.7.92 when Bob and Anne Warin were both killed in a car crash near Salisbury, while they were travelling to the Bournemouth Meeting of the British Association of Dermatologists. Bob was a Past-President of the B.A.D., and he and Anne had been such central figures in the Association for so many years that it was difficult to believe the Meeting could continue in their absence. The pall which the tragic news cast over that Meeting testifies to the tremendous affection and respect which British dermatologists had for Bob and Anne.

Bob, the son of a chemistry teacher, was born in Tadcaster on 19th December 1915 and was educated at St. Peter's School, York. He went on to Leeds University Medical School, where he was Student President of the Medical Society, and graduated in 1939. He obtained his M.D. in 1941, and then served as a Major in the R.A.M.C. (167 Field Ambulance) in North Africa and the Middle East from 1941 to 1946. During this time he worked as a general physician, gaining valuable experience with infectious diseases such as typhoid, and he took the MRCP examination in Cairo under special war-time regulations. After the war, he returned to Leeds General Infirmary and worked as Registrar in Dermatology under Professor Ingram until 1949, when he was appointed as Consultant Dermatologist (with Clifford Evans) at Bristol Royal Infirmary.

His clinical skills, his enthusiasm for his work, and his outstanding personal qualities of wisdom, kindliness and cheerfulness ensured that he was greatly in demand as an NHS consultant until his retirement in 1981. He also built up an extensive private practice which continued until the day of his death, aged 76, and patients were referred to him from all over the country, particularly for advice about the management of angio-oedema and urticaria, in which he was an authority. His patients loved him, and many wept when they were told of his death.

Despite this heavy clinical work-load, Bob was always active in research, particularly in the field of urticaria, and he was greatly in demand as a speaker at international meetings. In 1974, with R.H.Champion as co-author, he published 'Urticaria', a monograph which became the standard work on the subject, and he contributed a chapter to a large multi-author textbook on Immunology. He continued his work on urticaria after his retirement, with a weekly research clinic at BRI and at the time of his death he was working on a paper on the significance of a transient deficiency of C1 esterase inhibitor. He also published many excellent case-reports over the years, and younger members of the BRI Dermatology Department searching the literature on some arcane topic such as plasma cell granuloma of the lip, or reticulohistiocytosis as a marker of malignancy, were often surprised to find that the landmark paper was written by Bob in the 1950s.

Bob also played an important role in teaching, administration and medical politics. He was a founder member of the South-West of England and Wales Dermatological Society, which he organised 'single-handed' for many years, and which still flourishes. He was a successful President of the Bristol Med-Chi Society, and he was President of the British Association of Dermatologists in 1976. Many B A.D. members recall the Bristol Meeting as one of the most enjoyable ever held, largely because of Bob's personality and the tremendous support he had from Anne with the 'social programme'.

The BRI Dermatology Department flourished and was surely one of the happiest in the country under his benevolent leadership. He had a wonderful knack of getting people to give of their best for him, and in return he was always extremely supportive of his colleagues and junior staff. His paternal interest in his staff was so great that on one occasion he was overheard to suggest to his Senior Registrar, herself a mother of 3 children, that she should go to spend a penny before they set off together to a meeting.

Although his contribution to dermatology was enormous, Bob had many other interests. As a young man he played hockey for the Combined Universities and for Yorkshire and Gloucestershire, and he remained a keen cricketer throughout his life. Long after retirement he played for a Dowling Club team against trainee dermatologists, and was highest run scorer of the match. Similarly, at the BAD Meeting in Cambridge, he and Anne won the punt race by several lengths, despite strong competition from Oxbridge graduates more than 30 years his junior. On another occasion at an overseas conference, he came out of a restaurant around midnight in a jovial frame of mind and, for a bet, turned a series of elegant cart-wheels on the pavement. This would have been a good performance from anyone, but he was over 70 at the time. He missed only one day's work through illness from 1944 to 1992.

Gardening was his passion. He had an encyclopaedic knowledge of horticulture, which he loved to share with his friends, and his large garden in Clifton, which he occasionally opened to the public, was always a delight. He was on the Council of Bristol Zoo for over 25 years, and gave valuable advice with regard to the Zoo's wonderful gardens. Of course he provided dermatological expertise for ailments such as warts in white tigers, and fungal disease in rhinos, but he would also help out with more general problems such as difficult deliveries in giraffes.

He was a Governor of Clifton High School for Girls, and he and Anne were prominent in Clifton 'social life', including church and Clifton College activities. Both Bob and Anne had great charm with a gift for putting people at their ease, and since they knew almost everyone in Bristol, their presence at a cocktail party would ensure its success.

Bob was a great family man, and he derived strength and considerable support from Anne, who provided a role-model for many young wives to emulate. Their five children all went to University (their son Will is now a local G.P.), and in latter years their grand-children kept them busy, especially in the holidays (a recent 'fourth day of Christmas' when the ladies had all gone shopping and Bob was left in charge of approximately 10 grand-children, was one of the few occasions when I heard him admit to feeling slightly tired).

Once the fifth child had left home, Anne completed her formal education, obtaining a first class Honours degree in Humanities from the Open University. She then proceeded to write a series of books, including 'Dear Girl' about her father's experiences in the Royal Flying Corps in World War I, and 'Hilda' a historical novel based on the life of an Anglo-Saxon saint who became Abbess of Whitby. She also collaborated with Bob on a short book about Bristol Zoo, which became a best-seller, and their most recent publication, on the history of Litfield House (now the Private Medical Centre), was well-received by Bristol doctors. Anne's biography of St. Wilfred, a companion volume to 'Hilda', will be published in 1993.

J.L.Burton

## Figures and Tables

**Figure f1:**